# Experimental Cigarette Smoking by Domestic Fowls

**DOI:** 10.1038/bjc.1955.47

**Published:** 1955-09

**Authors:** P. R. Peacock

## Abstract

**Images:**


### EXPERIMENTAL CIGARETTE SMOKING BY DOMESTIC FOWLS.

P. R. PEACOCK.

*From the Cancer Research Department, Royal Beatson Memorial Hospital, Glasgow.*

Received for publication May 31, 1955.

EVIDENCE of an association between lung cancer and a history of heavy cigarette smoking has been reported both in Great Britain and in the United States of America (Doll and Hill, 1954 ; Wynder and Graham, 1950). It does not follow that heavy smoking necessarily entails an increased risk of lung cancer, since there may be great differences in individual susceptibility to the causal factors concerned, whose nature has not yet been established. Moreover, residence in towns seems also to be a possible aetiological factor in men who have lung cancer. Thus cigarette smoke and atmospheric pollution are extrinsic factors that should be investigated for possible carcinogens or co-carcinogens.

Experimental smoking by animals seems to be an obvious method of testing for carcinogenic agents in cigarette smoke, but there is great difficulty in devising a technique that resembles the human habit. Mice or other animals enlcosed in a confined space into which cigarette smoke is blown resemble non-smokers in a smoking compartment rather than smokers, and, like them, seem to find the experience disagreeable. Such animals are unable to breathe relatively fresh air between puffs of smoke as is done by human smokers, and must have their fur impregnated with smoke products which they lick when grooming themselves. Thus such experiments cannot be said to resemble closely the human habit.

The desirable criteria for a cigarette smoking test seem to be (*a*) that the smoke should be drawn into the lung of the animal without traversing a free air space in which cooling and precipitation of substances may occur ; (*b*) that the inhalation should be intermittent with access to relatively smoke-free air between puffs as in human smoking ; and (*c*) the distance travelled by the smoke between the cigarette and the lung should be about 12 inches as in the human subject. These criteria can be met by using the air sacs of fowls as a means of access to their lungs.

The anatomy of the bird's lung differs considerably from that of mammals, but serves the same physiological function and thus seems a suitable test for exposure to smoke. Moreover, spontaneous lung cancer seems to be rare in birds, in which, however, we were able to induce bronchial carcinoma with 2-acetyl laminofluorene (Peacock and Peacock, 1954). The existence of air sacs has been known for a very long time and many physiological studies have been made of the respiration of birds (Graham, 1939 ; McLeod and Wagers, 1939). The available evidence shows that most of the respiratory exchange in birds occurs during expiration when the air drawn through the bronchi into the air sacs during inspiration is passed back through the recurrent bronchi into the air passages

30

of the lungs. Thus smoke introduced into an air sac must traverse the lung before emerging through the mouth.

Preliminary injection of a liquid plastic which polymerises within 24 hours, through the trachea of freshly killed birds permitted a detailed study of the air sacs to be made. The transparent casts of abdominal air sacs, are illustrated in Fig. 1. There are also sacs in the axillary region running through the shoulder joint into the shaft of the humerus. They are well seen during the dissection of the injected specimen, but are more difficult to illustrate because of their tortuous course which makes the casts fragile and difficult to preserve.

After vigorous flapping of the wings the axillary air sacs can be seen pulsating just beneath the skin in the axilla over the shoulder joint. At this point an opening 5 mm. long was made, under ether anaesthesia, and the cut edges of the air sac were stitched to the skin with fine cotton thread. The resulting stoma was used four days later for introducing a glass canula attached to a simple smoking apparatus as illustrated (Fig. 2 and 3). This consists of an all-glass 20 ml. syringe and a Y-shaped pyrex glass tube with glass non-return valves in the two limbs not connected to the syringe. A cigarette inserted into one limb of the apparatus can thus be smoked by means of the syringe used as a pump.

The rate of smoking and the volume of each puff can easily be controlled by the operator. After some trials a routine was established as follows: 15 ml. of smoke are drawn slowly into the syringe so that the cigarette end just glows (in diffuse daylight). The smoke is then expelled steadily through the stoma into the lung of the fowl whence it emerges a second or two later through the mouth (Fig. 4). The bird is allowed to take five breaths before a second puff is injected in the same way and this process is repeated until fifty breaths have been taken. Thus on average ten injections each of 15 ml. of smoke are used during the smoking session. In this way the bird breathes, in and out, smoke with intermittent free air breathing as in human smoking.

At first exposure all birds seemed to experience malaise similar to that experienced by human smokers, and there seemed to be some variation in the severity of the symptoms. Some birds merely showed surprised interest in the smoke emerging from their beaks; others closed their eyes and drooped their heads, evidently disliking the experience. When this happened the exposure was stopped for the day.

Birds quickly became accustomed to the procedure and after a few days showed no signs of discomfort and accepted their smoke without opposition.

---

EXPLANATION OF PLATE.

FIG. 1.—White Leghorn hen. Trachea, main bronchi, lungs and abdominal air sacs injected with plastic. Viewed from behind. The abdominal viscera have been removed.

FIG. 2.—White Leghorn hen No. 4317, showing stoma (indicated by arrow) from left axilla to thoracic air sac.

FIG. 3.—All-glass pumping system for smoke injection: (i) and (ii) ground glass non-return valves; (iii) 20 ml. all-glass syringe; (iv) No. 19 stainless steel serum needle. With this apparatus no preliminary operation is necessary, as smoke can be injected directly through the skin into the thoracic air sac in the axilla.

FIG. 4.—White Leghorn fowl No. 4317. Smoke is emerging from the beak during injection through the stoma shown in Fig. 2.

1

2

3

4

Owing to a tendency for the stoma to heal after about fourteen days, a simpler procedure is now employed. A short, wide-bored, stainless steel needle (No. 19 serum) fitted to the outlet of the smoking apparatus is thrust directly into the axillary air sac through the skin. When smoke is drawn into the syringe it can be seen oscillating back and forth in the Y-tube with each breath if the needle is in the sac ; thus the risk of causing surgical emphysema can be avoided when injecting the smoke. At the time of writing four birds have been smoking half a cigarette each every other day for three months with no obvious ill effect. This is taken only as evidence that the dose is not immediately toxic and that the experiment can be carried on for a reasonably long period. As domestic fowls have lived for up to ten years in our experimental farm laboratory, it is probable that the test will have to be continued for several years before the effects can be assessed.

### SUMMARY.

A simple procedure for injecting cigarette smoke into the thoracic air sacs of fowls is described.

The smoke passes through one lung and out through the mouth and is partly re-inhaled, so that the whole respiratory system is exposed to the smoke.

The smoke inhalation is intermittent as in the human habit. Tolerance is quickly established and maintained at half a cigarette per bird every other day.

### REFERENCES.

Doll, R. and Hill, A. B.—(1954) *Brit. med. J.*, i, 1451.
Graham, J. D. P.—(1939) *J. Physiol.*, **97**, 133 and 525.
McLeod, W. M. and Wagers, R. P.—(1939) *J. Amer. vet. med. Ass.*, **95**, 59.
Peacock, P. R. and Peacock, A.—(1954) *Brit. J. Cancer*, **8**, 147.
Wynder, E. L. and Graham, E. A.—(1950) *J. Amer. med. Ass.*, **143**, 329.


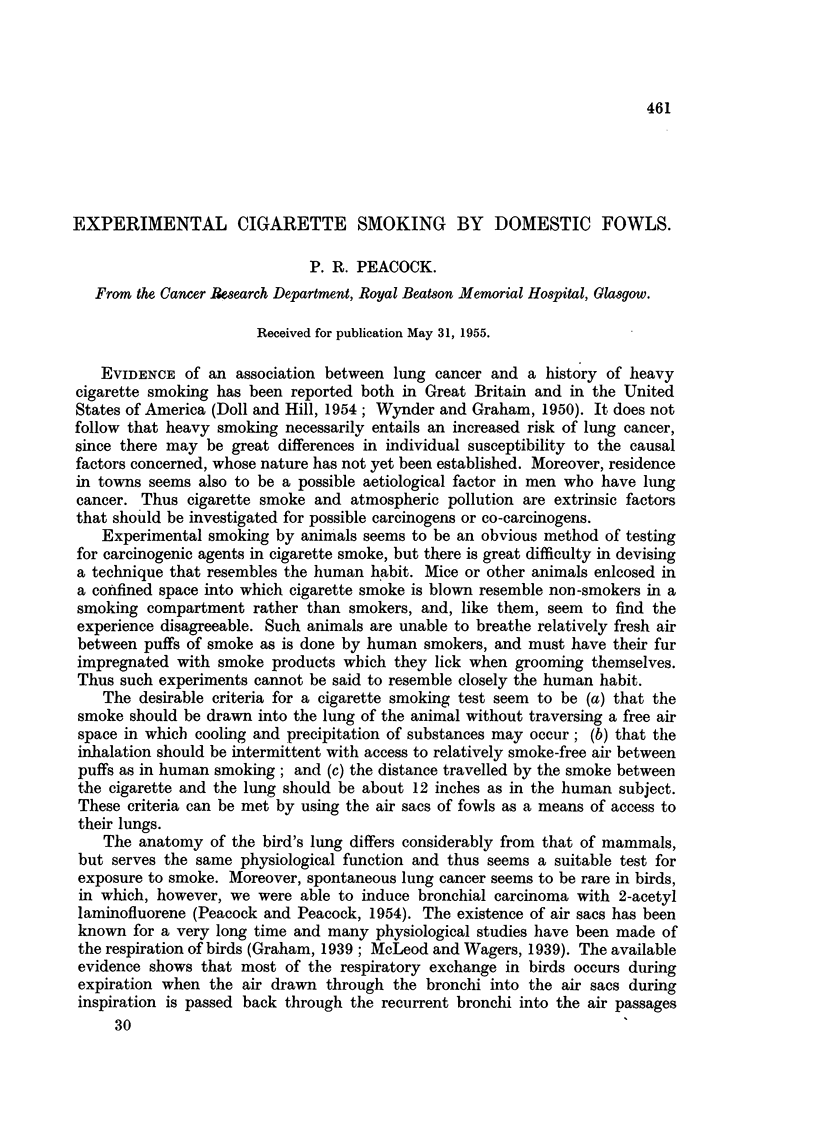

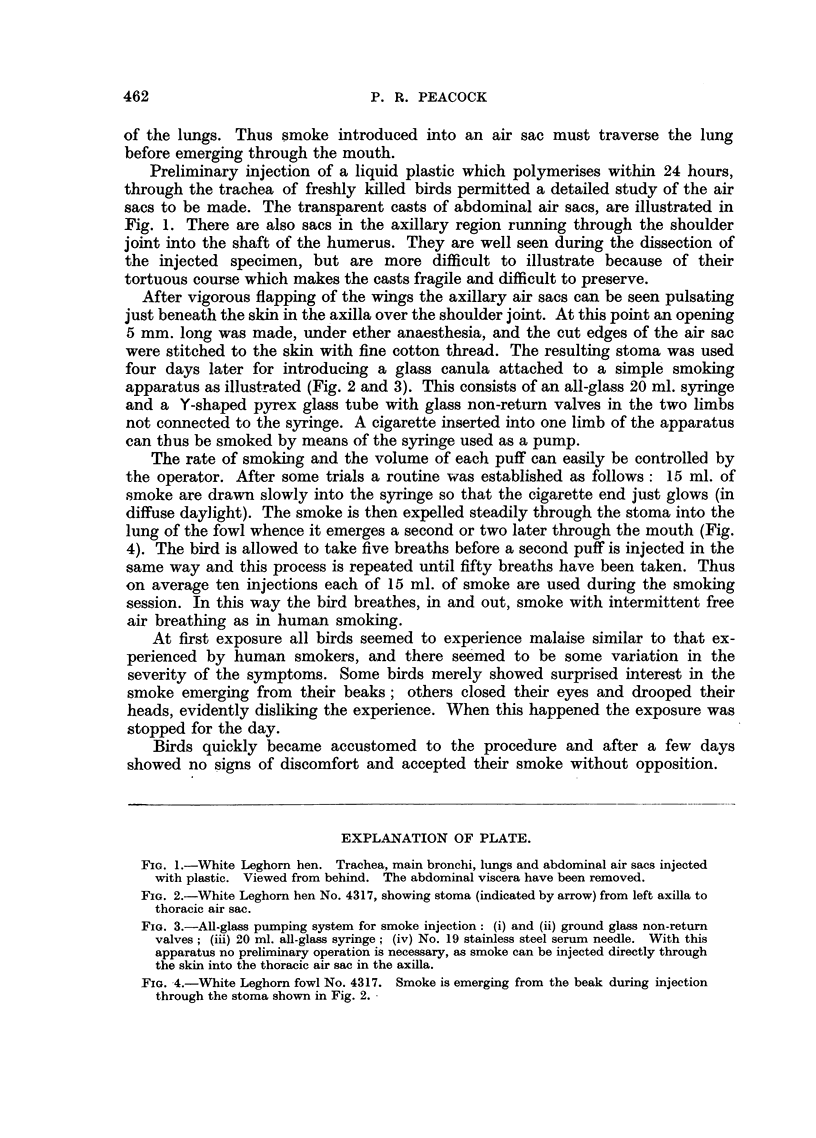

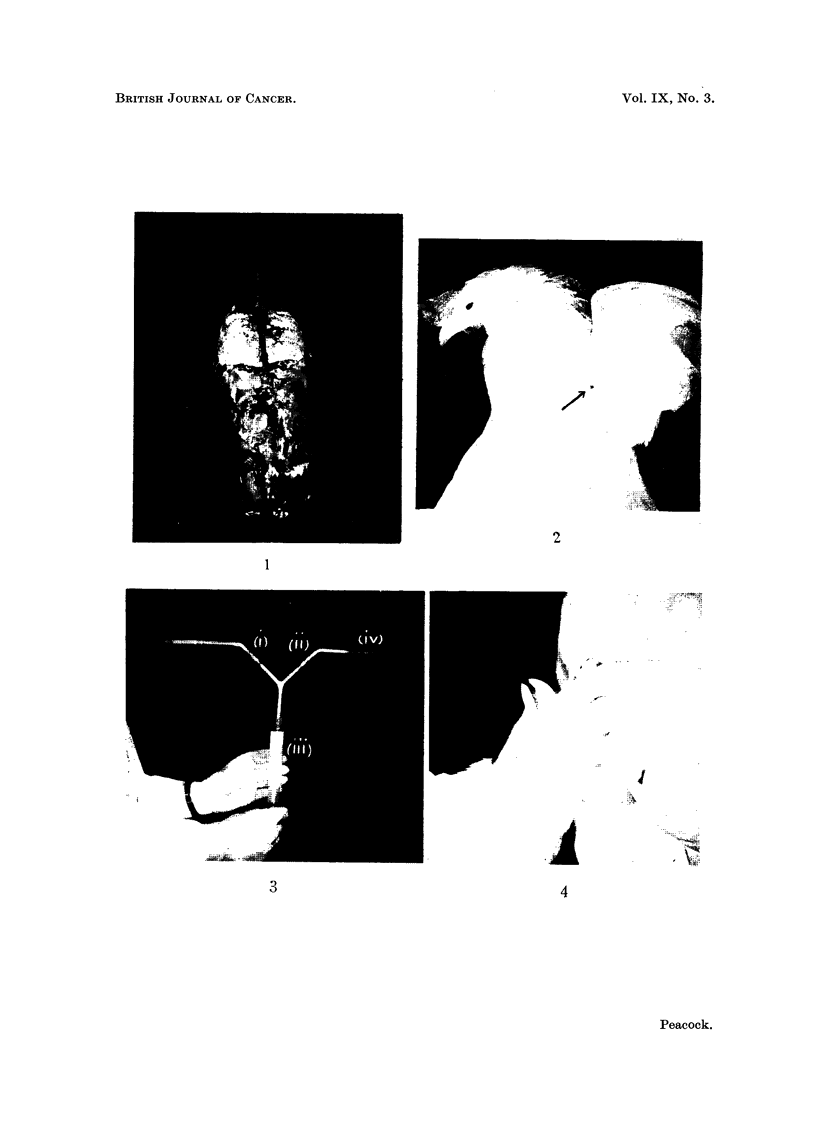

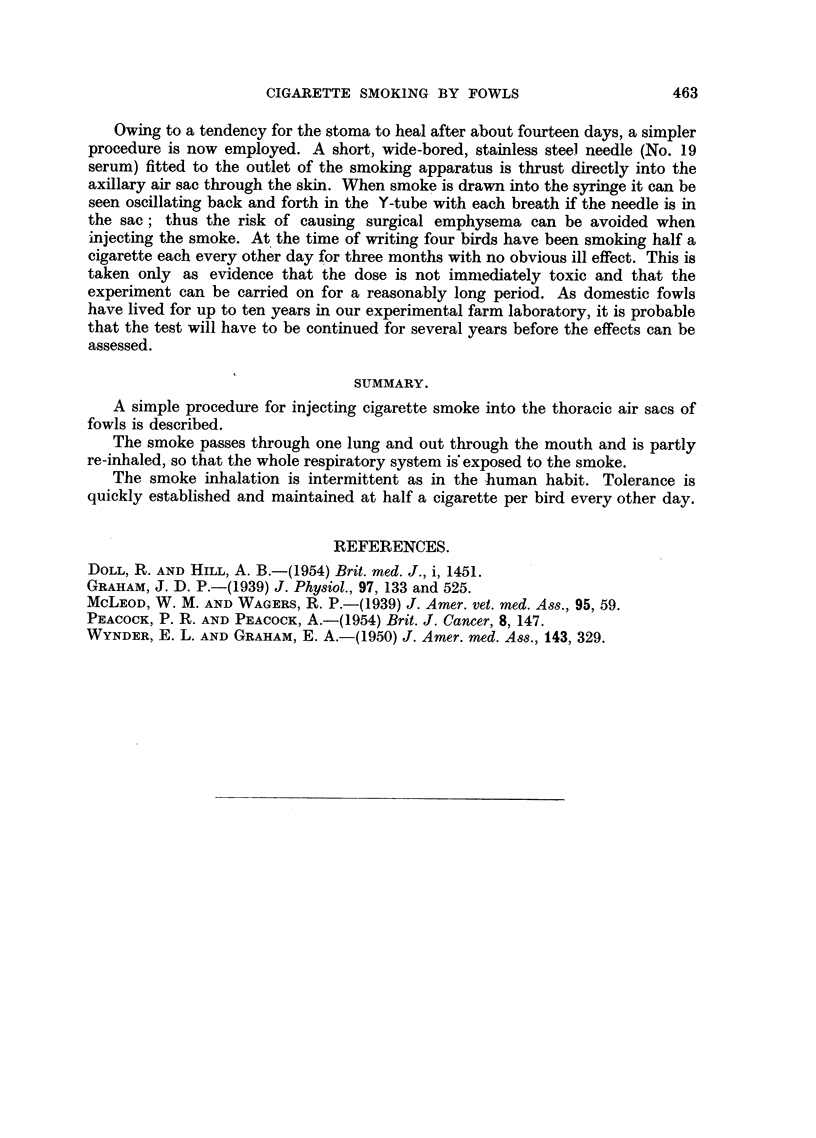


## References

[OCR_00168] Graham J. D. (1939). The air stream in the lung of the fowl.. J Physiol.

[OCR_00171] PEACOCK P. R., PEACOCK A. (1954). Epithelial tumours in fowls induced by 2-acetylaminofluorene.. Br J Cancer.

[OCR_00173] WYNDER E. L., GRAHAM E. A. (1950). Tobacco smoking as a possible etiologic factor in bronchiogenic carcinoma; a study of 684 proved cases.. J Am Med Assoc.

